# Supplementary material for: Oxygen Vacancies-Rich S-Cheme BiOBr/CdS Heterojunction with Synergetic Effect for Highly Efficient Light Emitting Diode-Driven Pollutants Degradation
Source: Nanomaterials (Basel). 2023 Feb 23;13(5):830. doi: 10.3390/nano13050830 (PMC10005353; doi:10.3390/nano13050830)
Supplement: Supplementary file 1 [file nanomaterials-13-00830-s001.zip › nanomaterials-2216437-supplementary.pdf]

Supplementary Materials

Oxygen Vacancies-Rich S-Cheme BiOBr/CdS Heterojunction with Synergetic Effect for Highly Efficient Light Emitting Diode-Driven Pollutants Degradation

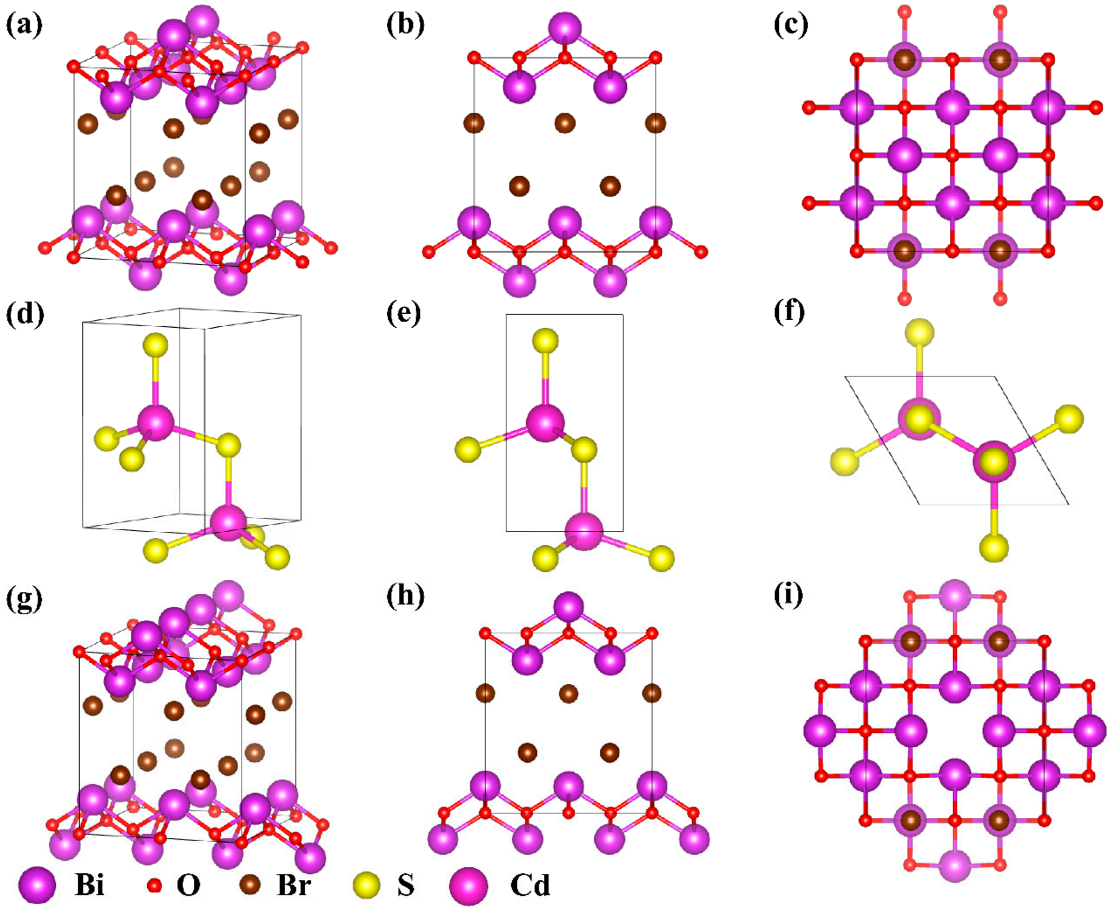

Figure S1. Standard oriented view (a) Front view (b) and top view (c) of the simulated structures of BiOBr; Standard oriented view (d) Front view (e) and top view (f) of the simulated structures of CdS; Standard oriented view (d) Front view (e) and top view (f) of the simulated structures of Vo-BiOBr.

Table S1 The surface fraction of O element by XPS spectra.

Sample	O-H	Oxygen vacancy	Bi-O
BiOBr	533.03 (1.55)	531.53 (6.36)	529.83 (92.09)
Vo-BiOBr	532.33 (1.03)	530.83 (44.74)	529.38 (54.26)
Vo-BiOBr/CdS	532.08(13.98)	530.88(46.50)	529.48(39.52)

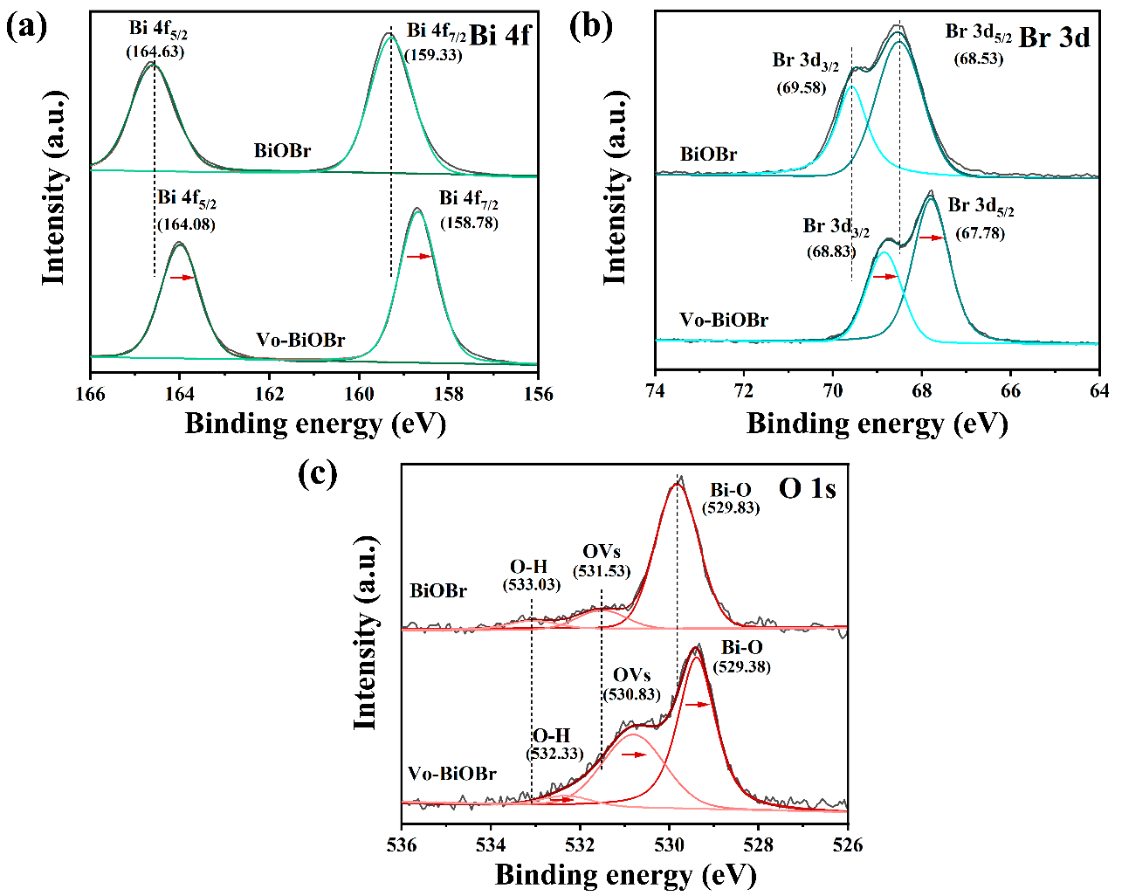

Figure S2. High-resolution XPS spectra of Bi 4f (a), Br 3d (b), O 1s (c) of BiOBr and Vo-BiOBr.

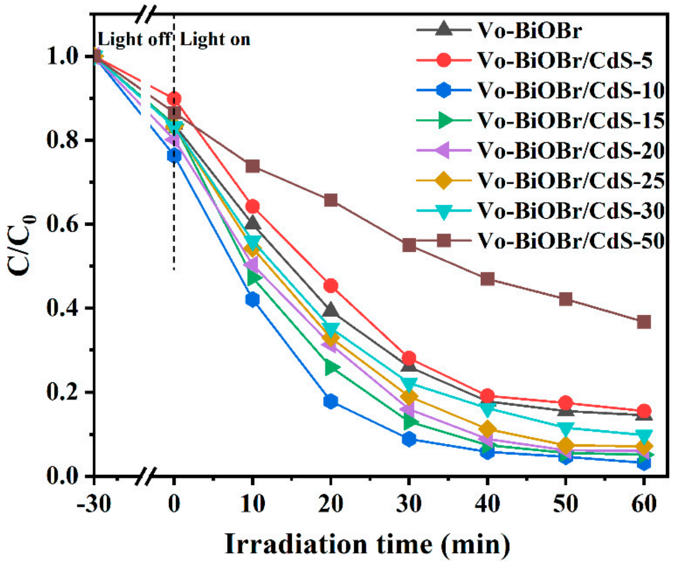

Figure S3. The removal efficiency of RhB over Vo-BiOBr/CdS with different mass ratio.

Table S2 Comparison of the photocatalytic activity of Vo-BiOBr/CdS with other related catalysts reported in the literature.

Catalyst	Experimental conditions Dosage; irradiation time; [pollutant]	Light source	Removal; Rate (%)	Reference
Vo-BiOBr/CdS	0.5 g/L 60 min<br>RhB=10 mg/L<br>MB=20 mg/L	LED lamp<br>(5 W)	97<br>94	This work
$BiVO_4$/BiOBr	1 g/L 180 min<br>RhB=15 mg/L	Xe lamp<br>(500 W)	90	[1]
BiOBr/ZnO	1 g/L 130 min<br>RhB=5 mg/L	Xe lamp<br>(300 W)	95	[2]
BiOCl/BiOBr	1 g/L 360 min<br>MB=10 mg/L	LED lamp	93	[3]
BiOBr/$BiPO_4$	1 g/L 120 min<br>RhB=15 mg/L	LED lamp<br>(12 W)	95	[4]
Bi_2MoO_6/BiOBr	1 g/L 40 min<br>MB=20 mg/L	LED lamp<br>(50 W)	90	[5]
BiOBr-Sn	0.2 g/L 120 min<br>RhB=10 mg/L	Xe lamp<br>(500 W)	70	[6]

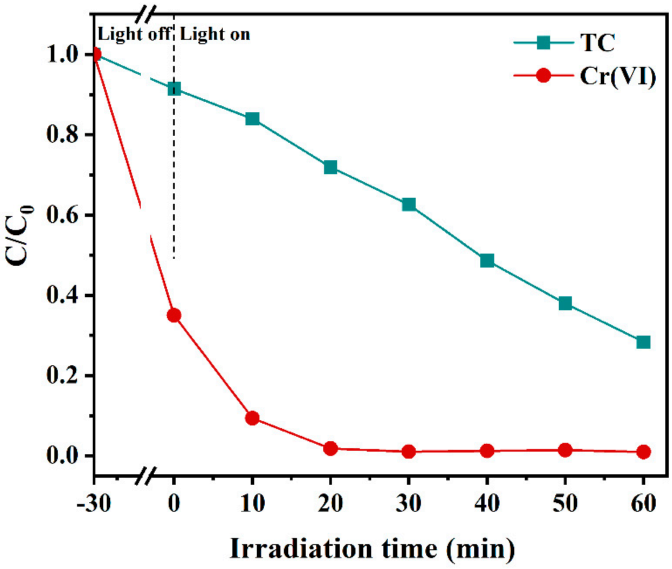

Figure S4. The removal efficiency of TC and reduction efficiency of Cr^{6+} over Vo-BiOBr/CdS under the irradiation of LED light.

Reference

1. Liu, S.; Chen, J.; Liu, D.; Shan, L.; Zhang, X. Improved visible light photocatalytic performance through an in situ composition-transforming synthesis of $BiVO_4$/BiOBr photocatalyst. *J Nanopart Res.* **2019**, *21*, 1-10.
2. Xing, Y.; He, Z.; Que, W. Synthesis and characterization of ZnO nanospheres sensitized BiOBr plates with enhanced photocatalytic performances. *Mater Lett.* **2016**, *182*, 210-213.
3. Zhang, J.; Lv, J.; Dai, K.; Liang, C.; Liu, Q. One-step growth of nanosheet-assembled BiOCl/BiOBr microspheres for highly efficient visible photocatalytic performance. *Appl. Surf. Sci.* **2018**, *430*, 639-646.
4. Zhao, H.j.; Wu, R.J.; Wang, X.C.; An, Y.m.; Zhao, W.X.; Ma, F. Heterojunction of $BiPO_4$/BiOBr photocatalysts for Rhodamine B dye degradation under visible LED light irradiation. *J Chin Chem Soc-Taip.* **2020**, *67*, 1016-1023.
5. Hu, T.; Yang, Y.; Dai, K.; Zhang, J.; Liang, C. A novel Z-scheme Bi_2MoO_6/BiOBr photocatalyst for enhanced photocatalytic activity under visible light irradiation. *Appl. Surf. Sci.* **2018**, *456*, 473-481.
6. Tu, X.; Qian, S.; Chen, L.; Qu, L. The influence of Sn (II) doping on the photoinduced charge and photocatalytic properties of BiOBr microspheres. *J Mater Sci.* **2015**, *50*, 4312-4323.
